# Epidemiology and impact of influenza in Mongolia, 2007–2012

**DOI:** 10.1111/irv.12268

**Published:** 2014-07-09

**Authors:** Alexanderyn Burmaa, Taro Kamigaki, Badarchyn Darmaa, Pagbajabyn Nymadawa, Hitoshi Oshitani

**Affiliations:** a National Influenza Center, National Center of Communicable DiseasesUlaanbaatar, Mongolia; b Department of Virology, Tohoku University Graduate School of MedicineSendai, Japan; c Mongolian Academy of Medical SciencesUlaanbaatar, Mongolia

**Keywords:** Epidemiology, impact, influenza, Mongolia, surveillance

## Abstract

**Background:**

Mongolia's Health Service began to conduct surveillance for influenza in the 1970s. This surveillance has become more comprehensive over time and now includes 155 sentinel sites in Mongolia. In this study, we analyzed the epidemiological characteristics and impact of influenza using data from influenza surveillance in Mongolia.

**Materials and methods:**

The data were collected by the National Influenza Center, Mongolia (NIC). Incidence rates of influenza-like illness (ILI) and severe acute respiratory infections (sARI) were calculated as the proportion of the number of ILI and sARI cases to the total population in the studied areas. Nasopharyngeal samples were collected and tested using real-time reverse transcription polymerase chain reaction [(rt)-RT-PCR]. Selected samples negative for influenza were tested for other respiratory pathogens by multiplex rt-RT-PCR.

**Results:**

Averages of 14·0 ILI and 0·8 sARI episodes per 100 population per year were observed during the five influenza seasons. The highest incidences of influenza associated with ILI and sARI were observed among children 0–4 years old. The number of ILI cases showed a clear seasonality, generally peaking between December and February. In contrast, sARI incidence peaked twice during each season. Influenza B was most prevalent during 2007–2008 and 2011–2012, influenza A (H3N2) during 2010–2011, seasonal A (H1N1) during 2008–2009, and A (H1N1) pdm09 during 2009–2010.

**Conclusions:**

Additional data on the epidemiology and impact of influenza including socioeconomic impact and vaccine effectiveness are required to develop a national influenza control policy, including a vaccination strategy. Our results provide useful data for developing such a policy.

## Introduction

Influenza viruses are highly infectious and cause seasonal epidemics. On average, the world's population experiences 3–5 million cases of severe illness and approximately 250 000–500 000 deaths each year because of influenza.^1^ These seasonal epidemics are caused by the accumulation of mutations in the antigenic sites of circulating seasonal influenza viruses, that is, antigenic drift. However, antigenic shift is another mechanism of antigenic change by influenza A. Antigenic shift occurs when an antigenically distinct virus emerges in the human population. Such a virus has the potential to cause a widespread global epidemic with high morbidity and mortality. Seasonal influenza epidemics generally occur during the winter in temperate climates, but the transmission of influenza may occur throughout the year in the tropics.^2^ Although the epidemiology and impact of influenza are well defined in developed countries, data in developing countries are still limited.^3^,^4^ Because of a lack of appropriate data on the epidemiology and impact of influenza, influenza control programs, including vaccination, are not a priority in most developing countries.^5^

Mongolia's Health Service began to conduct surveillance for influenza in the 1970s. The surveillance initially included reporting the number of cases with influenza-like illness (ILI) and some laboratory confirmation only in the capital city, Ulaanbaatar. This surveillance has become more comprehensive, involving more aimags (provinces) of the country. Major influenza epidemics with high morbidity were recorded during the 1980s and 1990s.^6^ During the country's political and economic transition in the 1990s, the public health infrastructure was fragmented followed by a subsequent reduction in ILI incidence. This reduction was likely because of both decreased reporting and a declining child population, which traditionally have the highest incidence of seasonal influenza.^7^ At present, influenza surveillance in Mongolia is coordinated by the National Influenza Center (NIC). Since the new influenza surveillance system started during the 2004–2005 season, the NIC increased the number of sentinel surveillance sites with a total of 155 sentinel sites by October 2009.^8^ Beginning with the 2006–2007 season, the NIC included surveillance for severe acute respiratory infections (sARI) with 15 sentinel sites. This surveillance has been gradually extended to cover all provincial hospitals, district hospitals, and special hospitals in Ulaanbaatar and consists of 37 sites covering the entire country since 2009–2010. In this study, we analyzed epidemiological and virological surveillance data to define the epidemiological characteristics and impact of influenza in Mongolia during the 2007–2008 to 2011–2012 influenza seasons.

## Methods and materials

### Geographic and climatic background

Mongolia is the second largest landlocked country with the second lowest population density in the world (1·74 people/km^2^ in 2010). Despite such a low population density, influenza transmission occurs regularly.^5^ The climate of Mongolia is harsh with four distinct seasons.

### Study sites

**Figure 1:**
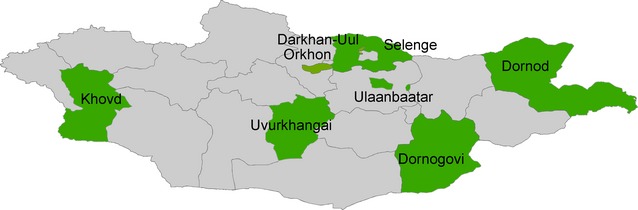
Location of sentinel sites for influenza surveillance in Mongolia since the 2009–2010 influenza season.

We analyzed both the ILI and sARI surveillance data from the 2007–2008 to 2011–2012 seasons to define the epidemiological characteristics of influenza in Mongolia. The Mongolian surveillance sites were divided into three categories to reflect different frequencies of specimen collection. The first category of influenza surveillance sentinel sites (ISSSs) reported ILI and sARI cases weekly and required collection of nasopharyngeal swabs from 5–10 patients with ILI and sARI per site and per week for the entire year. During peak influenza seasons, these sites collected specimens twice per week. The second category of ISSSs reported ILI and sARI cases weekly, but collected samples from patients only if there was a cluster or an unusual increase in the number of cases. The third category of ISSSs reported ILI and sARI cases weekly, but samples were collected only through consultation with the NIC and when the ILI rates exceeded a particular level (Figure1).

We only included data from the first category of ISSSs in this analysis. The first category of ISSSs has been chosen using following criteria: (i) representing different geographic regions, (ii) high population density, and (iii) along main railway route, which represents the whole population. These ISSSs were located in the regions of the following aimags: Dornogovi in the southeastern region, Dornod in the eastern region, Khovd in the western region, Selenghe in the northern region, and Uvurkhangai and Ulaanbaatar in the central region, where approximately one-third of the country's population lives. Each of these aimags has 4–5 family group practices (FGPs) in the aimag center (provincial capital) as ILI sentinel sites and one provincial hospital as a sARI sentinel site, except in Orkhon and Ulaanbaatar. Orkhon has 10 FGPs as ILI sentinel sites. Ulaanbaatar has 23 FGPs in eight districts as ILI sentinel sites and 13 hospitals, including eight district hospitals and five specialized hospitals, as sARI sentinel sites. Surveillance data collected in Darkhan-Uul and Orkhon, which represent Mongolia's second and third largest cities, respectively, were also included in this analysis. We used the ISSS vital statistical data from each year as the population data to calculate incidence rates. This vital statistical data have been submitted from aimag governments and reported annually by the National Health Center, Ministry of Health. A resident in each administrative area has a designated FGP as a local health provider depending the residential address. The incidence rates of ILI and sARI were calculated as the proportion of ILI and sARI cases to the total population in the area covered by surveillance. In this study, the peak period of influenza season was defined as the time when the number of ILI cases exceeded a threshold and the influenza virus positivity rates exceeded 15% or more among total tested samples in a week. The threshold was calculated by Serfling's method (cyclic regression model) 9 using nationwide data on ILI morbidity per 10 000 population from 2000 to 2011 in Mongolia.^10^

### Case definition

The World Health Organization (WHO) case definitions for ILI and sARI were used.^9^ Therefore, an ILI case was defined as a person with sudden onset of fever (>38·0°C), cough, and sore throat in the absence of another diagnosis. A sARI case was defined as a patient with ILI and shortness of breath or difficulty breathing, requiring hospitalization.

### Laboratory methods

Most of the laboratory tests for influenza viruses were conducted by the Virology Laboratory of the National Center of Communicable Diseases, Mongolia, which is a WHO-designated National Influenza Center. Aprroximately 3% samples were tested in branches established in Darkhan-Uul and Orkhon Provinces in 2010. Nasopharyngeal swab specimens were collected and immediately immersed into a sterile tube, containing virus transport medium, stored in refrigerator at the sentinel sites, and transported to the Virology Laboratory of the NIC or a branch laboratory once per week. The frequency of sample collection increased during the winter months. Samples were shipped by car from the central region, by plane from the western and eastern regions, and by train from the northern and southeastern regions.

The samples were initially inoculated on Madin–Darby canine kidney (MDCK) cells or embryonated hen's eggs to isolate the influenza viruses during the 2007–2008 and 2008–2009 seasons. The type/subtype of influenza virus was identified by reverse transcription polymerase chain reaction (RT-PCR) and the hemagglutination inhibition (HI) test. The reagents for the HI test were provided by WHO Collaborating Centers; National Institute of Infectious Diseases, Tokyo, Japan, and the Centers for Disease Prevention and Control (CDC), Atlanta, GA, USA.

We conducted the real-time (rt) RT-PCR assays to detect influenza viruses in randomly selected clinical samples in the 2008–2009 season, and it became a routine method for all samples received at the NIC beginning in August 2009. Assays were performed according to the standard protocol developed by the CDC,^11^ and primers were provided by WHO Collaborating Centers. Specimens positive for influenza A were further subtyped using specific rt-PCR analyses for seasonal A (H1N1), A (H3N2), and A (H1N1) pdm09. The influenza-positive specimens by (rt)-RT-PCR were inoculated on MDCK cells using a standard protocol.^12^ Multiplex rt-PCR was conducted for randomly selected samples beginning in the 2010–2011 season to detect other respiratory viruses, using commercially available multiplex PCR kits (FTD Respiratory Pathogens 21; FTD 2-96/12; Fast-track Diagnostics, Junglinster, Luxembourg). Selected influenza virus isolates were sent to the WHO Collaborating Center in Tokyo, Japan, for further analyses.

## Results

### Incidence of ILI and sARI

During the five influenza seasons from 2007–2008 to 2011–2012, an average of 14·0 ILI episodes per 100 population per year were recorded (Table1). The highest ILI incidence (22·2) was observed during the 2009–2010 season, which was 1·6 times higher than the average incidence for the five influenza seasons. An average of 0·8 sARI episodes per 100 population per year was observed (Table2). The highest sARI incidence (1·0) was observed during the 2009–2010 season, which was 1·3 times higher than the average incidence. The ratios of sARI to ILI episodes ranged from 0·045 during the 2009–2010 season to 0·066 during the 2011–2012 season, with an overall ratio of 0·054. The highest incidence was observed among children aged 0–4 years; 56·1% ILI cases and 77·1% sARI cases occurred in this age range. Because the age stratifications were different for ILI and sARI, it was not possible to directly compare incidences of ILI and sARI. However, the incidence of sARI in those aged >60 years was almost equal to that in children aged 5–9 years, whereas the incidence of ILI in those aged >65 years was only approximately one-fifth of that in children aged 5–9 years.

**Table 1:** Number of influenza-like illness (ILI) cases by influenza season and age group during the 2007–2008 and 2011–2012 seasons in Mongolia

Age group	2007–2008		2008–2009		2009–2010		2010–2011		2011–2012		Total	
					
	No. of ILI	Per 100	No. of ILI	Per 100	No. of ILI	Per 100	No. of ILI	Per 100	No. of ILI	Per 100	No. of ILI	Per 100
0–11 m	45 019	132·2	44 365	118·3	80 183	194·8	70 920	195·8	69 670	160·9	310 157	161·3
1–4 year	45 608	42·4	47 420	41·2	102 363	85·0	85 786	62·5	84 915	59·0	366 092	58·6
5–9 year	26 681	18·6	23 434	16·5	59 808	43·1	40 041	28·9	40 829	29·0	190 793	27·1
10–15 year	18 152	9·3	14 298	7·7	46 263	25·6	24 935	13·9	24 649	14·0	128 297	14·0
16–24 year	9979	3·2	8139	2·5	37 608	11·2	15 462	4·5	14 202	4·3	85 390	5·2
25–44 year	7331	1·4	6112	1·1	31 584	5·6	13 422	2·3	12 383	2·0	70 832	2·5
45–64 year	4451	1·8	3043	1·2	15 582	5·9	7236	2·6	7057	2·4	37 369	2·8
65 year <	2226	3·4	1473	2·1	6790	10·7	3550	5·2	3354	4·7	17 393	5·1
Total	159 447	9·8	148 284	8·9	380 181	22·2	261 352	14·7	257 059	14·1	1 206 323	14·0

**Table 2:** Number of severe acute respiratory infection (sARI) cases by influenza season and age group during the 2007–2008 and 2011–2012 seasons in Mongolia

Age group	2007–2008		2008–2009		2009–2010		2010–2011		2011–2012		Total	
					
	No. of sARI	Per 100	No. of sARI	Per 100	No. of sARI	Per 100	No. of sARI	Per 100	No. of sARI	Per 100	No. of sARI	Per 100
0–4 year	6775	4·8	6522	4·3	12 206	7·6	11 051	6·4	13 831	7·4	50 385	6·2
5–9 year	448	0·3	527	0·4	935	0·7	527	0·4	778	0·6	3215	0·5
10–19 year	625	0·2	631	0·2	1174	0·4	584	0·2	877	0·3	3891	0·2
20–49 year	602	0·1	550	0·1	2196	0·3	792	0·1	726	0·1	4866	0·1
50–59 year	223	0·2	169	0·1	410	0·3	261	0·2	249	0·2	1312	0·2
60 year <	288	0·3	237	0·2	505	0·5	356	0·3	337	0·3	1723	0·3
Total	8961	0·6	8636	0·5	17 426	1·0	13 571	0·8	16 798	0·9	65 392	0·8

### Detected influenza viruses by season

**Figure 2:**
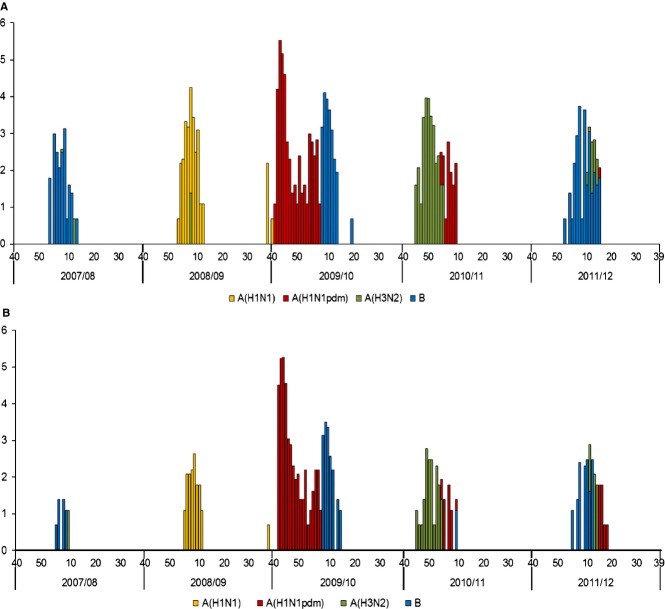
Temporal distribution of detected influenza viruses from (A) influenza-like illness (ILI) cases and from (B) severe acute respiratory infection (sARI) cases during the 2007–2008 and 2011–2012 seasons in Mongolia.

Figure2(A,B) presents the influenza viruses detected in ILI and sARI surveillance. The dominant influenza virus types/subtypes varied between seasons. Influenza B was the most commonly detected virus in the 2007–2008 and 2011–2012 seasons, influenza A (H3N2) in the 2010–2011 season, and seasonal A (H1N1) in the 2008–2009 season. A pandemic due to influenza A (H1N1) pdm09 occurred during the 2009–2010 season, and influenza A (H1N1) pdm09 was the predominant virus, followed by influenza B. Influenza virus-positive rates for both ILI and sARI were lower for the 2007–2008 and 2008–2009 seasons because the initial detection of influenza viruses was conducted by viral isolation, which is less sensitive compared with detection methods used later. Positive rates were highest for the 2009–2010 season because rt-PCR was used as the initial screening method for this season, and the number of samples and positive rates increased during the H1N1 pandemic in 2009.

### Temporal patterns of ILI and sARI cases

**Figure 3:**
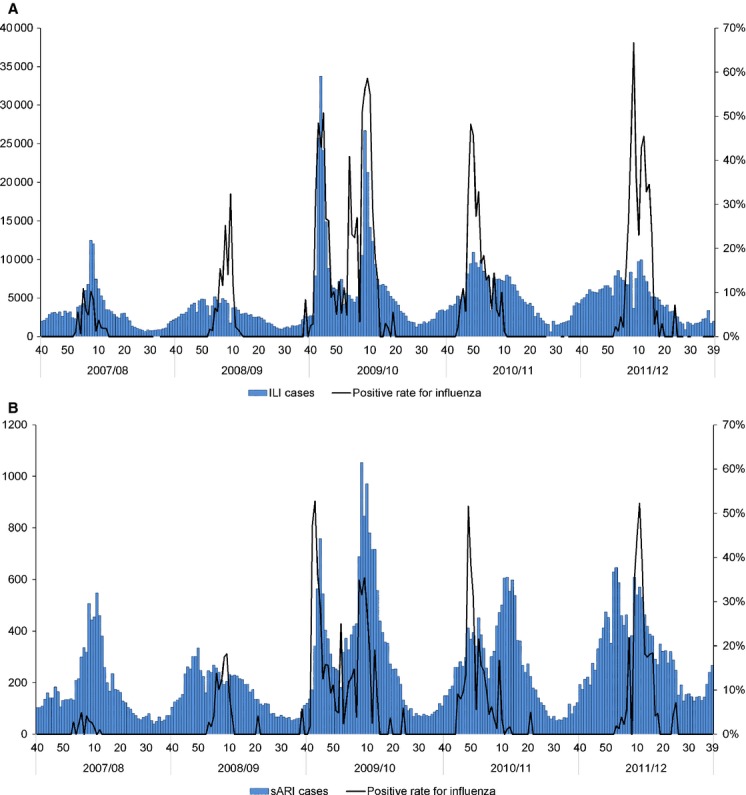
Temporal distribution of (A) influenza-like illness (ILI) cases and (B) severe acute respiratory infection (sARI) cases and influenza-positive rates for ILI and sARI cases during the 2007–2008 and 2011–2012 seasons in Mongolia.

Figure3(A) presents the temporal ILI trend and influenza positivity rates for the 2007–2008 and 2011–2012 seasons. The number of ILI cases showed clear seasonality peaking between December and February, except the 2009–2010 season when there were two peaks of ILI cases: one peak between October and November by A (H1N1) pdm09 and another in January 2010 by influenza B. However, an increase in the number of ILI cases was not necessarily associated with influenza activities in some seasons. ILI cases increased from approximately week 40 in 2008–2009, 2010–2011, and 2011–2012, but an increase in influenza virus-positive cases was only observed in January of the following year. ILI cases remained high after influenza activity subsided in the 2010–2011 season. A total of 606 randomly selected ILI specimens from the 2010–2011 and 2011–2012 seasons were tested for other respiratory viruses. Of these specimens, 271 (44·7%) were positive for at least one virus, including rhinovirus (RV) (63, 23·2%), respiratory syncytial virus (RSV) (34, 12·5%), human metapneumovirus (hMPV) (32, 11·8%), and human parainfluenza virus type 3 virus (hPIV3) (19, 7·0%). Figure S1 shows the monthly trend in influenza and other respiratory viruses among all cases. The early increase in ILI cases during the 2011–2012 season before the increase in the influenza-positive rate and the sustained number of ILI cases after the decrease in the influenza-positive rate during the 2010–2011 season may have been due to RV and RSV. Another sharp hMPV peak occurred after the influenza peak during the 2011–2012 season. Unlike other three respiratory viruses, RV has shown year-round circulation with two peaks before and after the increase in the influenza-positive rate during the 2011–2012 season.

Figure3(B) shows the weekly number of sARI cases and influenza-positive results. The sARI incidences showed slightly different patterns compared with those of the ILI data. Two peaks were generally observed during each season: the first peak occurred between October and December and the second one between January and March. The first peak was more severe including all age groups, but in the second peak, there was a slight increase of sARI incidence in children aged 0–4 years. The second peak during the 2009–2010 season, in which influenza B was the main influenza virus detected, had more sARI cases than in the first peak of A (H1N1) pdm09. Figure S2 shows the monthly trend of influenza and other respiratory viruses. A peak in RSV positive rates was observed during the second peak of the 2010–2011 and first peak of the 2011–2012 season, when extremely low influenza-positive rates occurred.

### Influenza virus positivity of ILI and sARI samples by age group

The overall influenza-positive rate was 10·6% for the ILI samples (Table S1a, b). Total influenza-positive rates by age group ranged from 5·1% to 13·1%. It was highest in children aged 5–9 years (13·1%) and lowest in those <1 year (5·1%) and ≥65 years (5·9%). The overall positive rate for sARI was identical to that for the ILI samples (10·6%) (Table S2a, b). The highest positive rate was observed in young adults aged 16–24 years (23·8%), and the lowest positive rate was observed in children aged 0–11 months (3·1%). Positive rates for different age groups were varied by type/subtype for both ILI and sARI. Positive rates for influenza A (H3N2) were the highest among children aged 1–4 years (ILI, 2·9%; sARI, 1·9%); positive rates for influenza A (H1N1) was the highest among children 5–9 years (ILI, 1·6%; sARI, 1·0%); and positive rates for influenza B was also the highest among children aged 5–9 years (ILI, 4·4%; sARI, 4·6%). However, age distribution of influenza A (H1N1)pdm09 was significantly different, and the highest positive rates were observed among young adult aged 16–24 years (ILI, 9·1%; sARI, 20·3%).

## Discussion

We analyzed the epidemiology and impact of influenza in Mongolia using national ILI and sARI surveillance data from 2007 to 2010 including the pandemic caused by influenza A(H1N1)pdm09. Accumulation of such data would be useful because the surveillance provides the basic data for the comparision of disease burden and severity of future influenza epidemics. Several studies regarding the epidemiology of influenza in Mongolia have been previously published.^13^–^15^ Two of these studies were limited by the scope of the population they examined and provided little information about the virus.^14^ A more recent study on influenza epidemiology in the Western Pacific Region provided more data on the temporal trends in both ILI and influenza-positive cases, but detailed analyses including age-stratified data were not available.^13^ Therefore, we believe that the present study provides more comprehensitve data on influenza epidemiology in Mongolia.

Children <5 years old had the highest incidence of both ILI and sARI during the study period. A community-based study in Mongolia also showed the highest incidence in young children.^16^ Similar results have been obtained in other countries.^17^–^20^ In addition, we observed a higher ratio of sARI to ILI among adults >60 years old, although the number of sARI cases in that age group remained low. The low number of ILI and sARI among adults may be because of the health-seeking behavior of this group; most elderly people with ILI symptoms may not visit an outpatient clinic unless they develop severe complications that require hospitalization. The higher proportion of sARI among the eldery indicates more severe influenza infection in this age group, which is compatible with other countries where severe complications are reportedly more common in the elderly.^21^–^23^

The prevelance of influenza virus types/subtypes varied between seasons, and the same virus subtype never predominated during more than two consecutive seasons. The dominant peaks for influenza viruses were observed between December and February, a pattern that has been observd in other countries in temperate regions.^24^ The rate of ILI incidence was the highest during the 2009–2010 season. This may have been because of the observation that more ILI cases visited healthcare facilities during the first outbreak of A (H1N1) pdm09. In addiiton, sARI incidence was highest during the 2009–2010 season. However, more sARI cases were observed during the second peak than during the first peak of the season. The first peak was primarily due to A (H1N1) pdm09, and the second peak was due to influenza B and RSV. Influenza B is believed to cause milder infection than influenza A viruses. However, recent studies in China and Hong Kong indicate a high disease burden for influenza B.^25^,^26^

ILI and sARI samples during the 2010–2011 and 2011–2012 influenza seasons were randomly selected to be tested for other respiratory pathogens. Our results indicated that the increase of ILI activity before and after influenza virus circulation may be due to other respiratory viruses, including RSV, hMPV, or hPIV3. These three viruses have seasonal epidemic peaks in children.^27^ A recent study reported that PIV, RSV, and hMPV cause outbreaks twice per year (one peak between October and December and another peak between March and May) in Mongolia.^28^ RSV had a considerably higher positive rate than influenza in hospitalized patients with sARI, as observed in other studies.^29^ This may be because of the finding that most sARI cases in the present study were observed in small children, and RSV is the predominant cause of sARI in this age group. After introducing rt-PCR as the initial screening method during the 2009–2010 influenza season, the influenza virus-positive rates significantly increased, as was also noted in a surveillance study from Thailand.^30^ The seasonality of influenza in temperate regions differs from that in the tropics. Influenza epidemics display a distinct seasonality in temperate regions, with widespread transmission typically occurring during the winter months: November–March in the Northern Hemisphere and May–September in the Southern Hemisphere.^24^ ILI activity in Mongolia demonstrates an increasing trend from approximately week 40, with a peak between December and February each year. This observation is consistent with data from neighboring countries, Russia and Northern China 31 and those from other countries and regions with temperate climates.^32^

### Limitations of the study

We have recognized several limitations of our study. Although we included weekly ILI and sARI data from seven provinces, along with eight districts in the city of Ulaanbaatar, laboratory testing results only included data from selected sentinel sites in these areas and may not have represented the pattern of virus circulation for the entire country. Also we could consider some false-negative results particularly when samples collected in latter in the course of illnesses were transferred and tested in the laboratory. This might have resulted in underestimation of the true influenza disease burden on ILI and sARI cases. The frequency of sample collection also differed between aimags because of a lack of transportation infrastructure. Although our surveillance included several healthcare facilities, it still depended on health-seeking behavior. More than half of ILI cases were children <5 years. This may reflect higher health-seeking behavior by this age group.

## Conclusion

This study provides the first comprehensive data on the epidemiology and impact of influenza in Mongolia, which were derived from ILI and sARI surveillance. The results demonstrate a clear seasonality of influenza events in Mongolia and the need to develop evidence-based control strategies for influenza.

## Supporting Information

**Figure S1.** Temporal distribution of influenza-like illness (ILI) cases and positive rates for influenza, rhinoviruses (RV), respiratory–syncytial viruses (RSV), human metapneumoviruses (hMPV), and human parainfluenza virus type 3 virus (hPIV3) during the 2010–2011 and 2011–2012 seasons in Mongolia.

**Figure S2.** Temporal distribution of severe acute respiratory infection (sARI) cases and positive rates for influenza, rhinoviruses (RV), respiratory–syncytial viruses (RSV), human metapneumoviruses (hMPV), and human parainfluenza virus type 3 virus (hPIV3) during the 2010–2011 and 2011–2012 seasons in Mongolia.

**Table S1a.** Number of influenza virus-positive samples by age group for influenza-like illness (ILI) cases in Mongolia during the 2007/8–2011/12 seasons. **Table S1b.** Number of influenza virus positive samples by season for ILI cases in Mongolia during the 2007/8–2011/12 seasons. **Table S2a.** Number of influenza virus positive samples by age group for severe acute respiratory infection (sARI) cases in Mongolia during the 2007/8–2011/12 seasons. **Table S2b.** Number of influenza virus positive samples by season for sARI cases in Mongolia during the 2007/8–2011/12 seasons.
