# Supplementary material for: Epidemiology and impact of influenza in Mongolia, 2007–2012
Source: Influenza Other Respir Viruses. 2014 Jul 9;8(5):530–7. doi: 10.1111/irv.12268 (PMC4181816; doi:10.1111/irv.12268)
Supplement: Supplementary file 3 [file irv0008-0530-SD3.docx]

Supplementary Table 1a. Number of influenza virus-positive samples by age group for influenza-like illness (ILI) cases in Mongolia during the 2007/8–2011/12 seasons

| Age group | Tested ILI samples | A(H1N1) | Positive rate | A(H1N1pdm) | Positive rate | A(H3N2) | Positive rate | B | Positive rate | Total influenza positives | Positive rate |
| --- | --- | --- | --- | --- | --- | --- | --- | --- | --- | --- | --- |
| 0-11 m | 958 | 1 | 0.1% | 8 | 0.8% | 16 | 1.7% | 24 | 2.5% | 49 | 5.1% |
| 1-4 y | 5,728 | 94 | 1.6% | 163 | 2.8% | 168 | 2.9% | 137 | 2.4% | 562 | 9.8% |
| 5-9 y | 2,879 | 46 | 1.6% | 150 | 5.2% | 53 | 1.8% | 127 | 4.4% | 376 | 13.1% |
| 10-15 y | 2,534 | 37 | 1.5% | 152 | 6.0% | 50 | 2.0% | 80 | 3.2% | 319 | 12.6% |
| 16-24 y | 1,829 | 17 | 0.9% | 166 | 9.1% | 20 | 1.1% | 31 | 1.7% | 234 | 12.8% |
| 25-44 y | 2,420 | 25 | 1.0% | 135 | 5.6% | 38 | 1.6% | 54 | 2.2% | 252 | 10.4% |
| 45-64 y | 1,295 | 6 | 0.5% | 33 | 2.5% | 21 | 1.6% | 24 | 1.9% | 84 | 6.5% |
| 65 y< | 269 | 2 | 0.7% | 2 | 0.7% | 7 | 2.6% | 5 | 1.9% | 16 | 5.9% |
| Total | 17,912 | 228 | 1.3% | 809 | 4.5% | 373 | 2.1% | 482 | 2.7% | 1,892 | 10.6% |

Supplementary Table 1b. Number of influenza virus positive samples by season for ILI cases in Mongolia during the 2007/8–2011/12 seasons

| Season | Tested ILI samples | A(H1N1) | Positive rate | A(H1N1pdm) | Positive rate | A(H3N2) | Positive rate | B | Positive rate | Total positives | Positive rate |
| --- | --- | --- | --- | --- | --- | --- | --- | --- | --- | --- | --- |
| 2007/08 | 5,032 | 1 | 0.4% | - | 0.0% | 35 | 9.4% | 97 | 20.1% | 133 | 7.0% |
| 2008/09 | 5,010 | 225 | 98.7% | - | 0.0% | 4 | 1.1% | - | 0.0% | 229 | 12.1% |
| 2009/10 | 3,316 | 2 | 0.9% | 724 | 89.5% | - | 0.0% | 217 | 45.0% | 943 | 49.8% |
| 2010/11 | 2,452 | - | 0.0% | 70 | 8.7% | 237 | 63.5% | 1 | 0.2% | 308 | 16.3% |
| 2011/12 | 2,102 | - | 0.0% | 15 | 1.9% | 97 | 26.0% | 167 | 34.6% | 279 | 14.7% |
| Total | 17,912 | 228 | 100.0% | 809 | 100.0% | 373 | 100.0% | 482 | 100.0% | 1,892 | 100.0% |

Supplementary Table 2a. Number of influenza virus positive samples by age group for severe acute respiratory infection (sARI) cases in Mongolia during the 2007/8–2011/12 seasons

| Age groups | Tested sARI samples | A(H1N1) | Positive rate | A(H1N1pdm) | Positive rate | A(H3N2) | Positive rate | B | Positive rate | Total influenza positives | Positive rate |
| --- | --- | --- | --- | --- | --- | --- | --- | --- | --- | --- | --- |
| 0-11 m | 2,256 | 5 | 0.2% | 23 | 1.0% | 18 | 0.8% | 24 | 1.1% | 70 | 3.1% |
| 1-4 y | 4,488 | 38 | 0.8% | 169 | 3.8% | 86 | 1.9% | 88 | 2.0% | 381 | 8.5% |
| 5-9 y | 586 | 6 | 1.0% | 75 | 12.8% | 6 | 1.0% | 27 | 4.6% | 114 | 19.5% |
| 10-15 y | 428 | 3 | 0.7% | 68 | 15.9% | 5 | 1.2% | 10 | 2.3% | 86 | 20.1% |
| 16-24 y | 867 | 4 | 0.5% | 176 | 20.3% | 4 | 0.5% | 22 | 2.5% | 206 | 23.8% |
| 25-44 y | 1,349 | 2 | 0.1% | 165 | 12.2% | 9 | 0.7% | 25 | 1.9% | 201 | 14.9% |
| 45-64 y | 515 | 3 | 0.6% | 48 | 9.3% | 5 | 1.0% | 2 | 0.4% | 58 | 11.3% |
| 65 y< | 115 | 0 | 0.0% | 4 | 3.5% | 1 | 0.9% | 2 | 1.7% | 7 | 6.1% |
| Total | 10,604 | 61 | 0.6% | 728 | 6.9% | 134 | 1.3% | 200 | 1.9% | 1,123 | 10.6% |

Supplementary Table 2b. Number of influenza virus positive samples by season for sARI cases in Mongolia during the 2007/8–2011/12 seasons

| Season | Tested sARI samples | A(H1N1) | Positive rate | A(H1N1pdm) | Positive rate | A(H3N2) | Positive rate | B | Positive rate | Total positives | Positive rate |
| --- | --- | --- | --- | --- | --- | --- | --- | --- | --- | --- | --- |
| 2007/08 | 2,065 | - | 0.0% | - | 0.0% | 5 | 3.7% | 16 | 8.0% | 21 | 1.9% |
| 2008/09 | 1,936 | 61 | 100.0% | - | 0.0% | 1 | 0.7% | 1 | 0.5% | 63 | 5.6% |
| 2009/10 | 3,479 | - | 0.0% | 679 | 93.3% | - | 0.0% | 117 | 58.5% | 796 | 70.9% |
| 2010/11 | 1,544 | - | 0.0% | 31 | 4.3% | 74 | 55.2% | 5 | 2.5% | 110 | 9.8% |
| 2011/12 | 1,580 | - | 0.0% | 18 | 2.5% | 54 | 40.3% | 61 | 30.5% | 133 | 11.8% |
| Total | 10,604 | 61 | 100.0% | 728 | 100.0% | 134 | 100.0% | 200 | 100.0% | 1,123 | 100.0% |
